# Longitudinal changes in personal recovery in individuals with psychotic disorders through hospitalisation in a psychiatric ward: preliminary findings

**DOI:** 10.1186/s12888-021-03347-3

**Published:** 2021-07-08

**Authors:** Norika Mitsunaga-Ohmuro, Noriyuki Ohmuro

**Affiliations:** 1 Department of Health Sciences, Tohoku University Graduate School of Medicine, Sendai, Miyagi Japan; 2 Department of Psychiatry, Osaki Citizen Hospital, Osaki, Miyagi Japan

**Keywords:** Clinical recovery, Hospitalisation, Inpatients, Longitudinal change, Personal recovery, Psychotic disorders, Schizophrenia, Self-efficacy, Self-esteem

## Abstract

**Background:**

This study aimed to investigate whether personal recovery indices in individuals with psychotic disorders would change through hospitalisation in a psychiatric ward and to identify factors associated with these changes.

**Methods:**

Participants underwent assessments for personal recovery using the Questionnaire about the Process of Recovery, Recovery Assessment Scale, and Self-Identified Stage of Recovery Part A and B; clinical symptoms using the Positive And Negative Syndrome Scale; self-efficacy using the General Self-Efficacy Scale; and self-esteem using the Rosenberg Self-Esteem Scale at baseline and before hospital discharge. Wilcoxon signed-rank tests were administered for longitudinal comparisons between baseline and follow-up. Spearman’s rank correlation tests were conducted to assess correlations of longitudinal changes in personal recovery with baseline values of personal recovery as well as baseline values or changes in the Positive And Negative Syndrome Scale, General Self-Efficacy Scale, and Rosenberg Self-Esteem Scale.

**Results:**

Thirty-four individuals with psychotic disorders completed the assessments. The average duration of the current hospitalisation was 81.9 days (SD, 15.3; median, 85.0; range, 51–128 days). No significant changes were observed in personal recovery, self-efficacy, and self-esteem, although clinical symptoms significantly improved. Significant correlations were found between positive changes in the Recovery Assessment Scale and improvements in negative symptoms; between positive changes in the General Self-Efficacy Scale and those in personal recovery assessed with the Questionnaire about the Process of Recovery, Recovery Assessment Scale, and Self-Identified Stage of Recovery part A; and between positive changes in the Rosenberg Self-Esteem Scale and those in the Self-Identified Stage of Recovery part B.

**Conclusion:**

This study revealed longitudinal relationships between changes in personal recovery and amelioration of negative symptoms or enhancement of self-efficacy and self-esteem through moderate length of hospitalisation in individuals with psychotic disorders. Considering the small sample size in this study, further studies with a larger sample size are needed to confirm the present finding.

**Trial registration:**

The protocol of this study is registered in the UMIN Clinical Trials Registry (UMIN-CTR; ID: UMIN000035131).

## Background

In the last decade, a paradigm shift has occurred in how to define ‘recovery’ for individuals with psychotic disorders, particularly schizophrenia. To date, recovery for these people has been focused mainly on remission of clinical symptoms and improvement of function. More recently, beyond ‘clinical recovery’, a concept of ‘personal recovery’ has been advocated and considered an important target for the care for these individuals [1–3]. For instance, Anthony defined personal recovery as ‘a deeply personal, unique process of changing one’s attitudes, values, feelings, goals, skills, and/or roles’ and ‘a way of living a satisfying, hopeful, and contributing life even with limitations caused by illness’ [1].

Since personal recovery is considered a unique process of an individual, achievement of personal recovery has often been discussed qualitatively using one’s own narrative [2]. In addition, many scales measuring how much one’s personal recovery is attained have been developed, and studies utilising such scales have been undertaken [4]. Many studies have examined factors that have cross-sectional relationships with degrees of personal recovery. A meta-analysis indicated that the severity of both positive and negative symptoms is negatively associated with degrees of personal recovery [5], and other studies have demonstrated that high self-efficacy [6] and high self-esteem [7] are positively correlated with degrees of personal recovery.

Notably, most studies focusing on personal recovery have been performed in outpatients [5, 8]; accordingly, findings of personal recovery of inpatients are relatively sparse. To promote personal recovery, it has been suggested that mental health care providers should minimise the application of forced intervention under involuntary treatment settings [9]. Nevertheless, admission to psychiatric wards is often inevitable for some individuals with severe symptoms. However, it remains unknown whether indices of personal recovery would change through hospitalisation.

Based on previous findings of cross-sectional relationships between indices of personal recovery and those of severity of clinical symptoms [5], self-efficacy [6], and self-esteem [7], we hypothesised that indices of personal recovery would improve through hospitalisation in a psychiatric ward, and improvements in personal recovery would be correlated with amelioration in clinical symptoms and reinforcement in self-efficacy and self-esteem. Accordingly, the current study investigated whether indices of personal recovery in individuals with psychotic disorders would change through hospitalisation in a psychiatric ward and to identify related factors associated with changes in indices for personal recovery.

## Methods

### Participants

The protocol of this study is registered in the UMIN Clinical Trials Registry (UMIN-CTR; ID: UMIN000035131 posted on 07/01/2019). This study was approved by the Ethics Committee of the Tohoku University Graduate School of Medicine (2018–1-655) and has been conducted according to the principles expressed in the Declaration of Helsinki. All participants provided written informed consent, and the data were anonymised. Participants were recruited between January 2018 and November 2019 from an acute treatment unit of three psychiatric hospitals located at Miyagi Prefecture, Japan. The number of beds in each treatment unit was 40 to 60, and the units were not specialised for treatment for individuals with psychotic disorders. Inclusion criteria were as follows: a diagnosis of psychotic disorders as per the Diagnostic and Statistical Manual of Mental Disorders, 5th edition [10], and age between 16 and 65 years. Exclusion criteria were as follows: severe physical conditions; comorbid diagnosis of substance-induced disorders, organic mental disorders, or intellectual disability; having difficulty in the comprehension of Japanese; or conditions under forced isolation or physical restraint. Those who had been admitted to the psychiatric ward were assessed by an in-charge psychiatrist, and subsequently, those who were judged to be eligible for participation in the study and provided informed consent were finally included in the current study. The offered intervention was organised by each in-charge psychiatrist based on the usual treatment regimen, which does not include a specific recovery-centred approach. Treatment regimens varied among the participants. Most participants were administered antipsychotics, and some participants were also medicated with other classes of psychotropic drugs and provided needs-based case management by in-charge psychiatrists and social workers. A very small number of participants were provided occupational therapy or social skills training by occupational therapists or clinical psychologists. No participants were provided structured psychotherapy, such as cognitive behavioural therapy, but all participants were cared for based on non-specific supportive psychotherapy. Participants underwent assessments at baseline and prior to discharge from the hospital. Baseline assessments were performed as soon as possible at admission in the ward after permission for inclusion in the study was provided by the participant and in-charge psychiatrist. Follow-up assessments were administered less than 1 month prior to discharge. Participants who decided to transfer to another hospital immediately after the discharge were excluded from the study.

### Assessments for personal recovery

As the main outcomes, the following four self-reported measures were used to assess the participants’ personal recovery.

### Questionnaire about the process of recovery

The Questionnaire about the Process of Recovery (QPR) was developed collaboratively with service user researchers [11, 12]. This measure consists of 22 items, and each item is rated on a 5-point Likert scale ranging from ‘strongly disagree (1 point)’ to ‘strongly agree (5 points)’. The summation of the scores was adopted as the main outcome measure (higher scores represents greater recovery). The QPR has been confirmed for its reliability and validity [6, 12] and involves all categories of the ‘CHIME’ framework (i.e., Connectedness, Hope and Optimism, Identity, Meaning and Purpose, and Empowerment), which are regarded as key factors for achieving personal recovery [4]. The Japanese version of the QPR, which has been confirmed for its reliability and validity [13], was used in the present study.

### Recovery assessment scale

The Japanese version of the Recovery Assessment Scale (RAS) [14], developed based on a shortened version of the RAS (24 items) [15] that was originated from a 41-item RAS [16, 17], and confirmed for its reliability and validity [14], was used in the current study. Each item was rated on a 5-point Likert scale ranging from ‘strongly disagree (1 point)’ to ‘strongly agree (5 points)’. The summation of the scores was calculated and adopted as the main outcome measure. Higher summation scores indicate greater recovery achievement.

### Self-identified stage of recovery part a and B

The Self-Identified Stage of Recovery Part A (SISR-A) was developed to evaluate a person’s achieved recovery stage based on a 5-stage recovery model, and Part B (SISR-B) was established to assess a person’s processes of recovery [18]. In the assessment of SISR-A, a participant needs to choose one of the five statements representing their achieved recovery stage, i.e., moratorium stage, awareness stage, preparation stage, rebuilding stage, and growth stage. One to five points are given corresponding to the chosen statement, and a higher score indicates a higher stage of achieved recovery. For example, in cases in which a participant chooses the ‘moratorium stage’ statement (‘I don’t think people can recover from mental illness. I feel that my life is out of my control, and there is nothing I can do to help myself’), 1 point is given; in cases in which the participant chooses ‘the growth stage’ statement (‘I feel I am in control of my health and my life now. I am doing very well and the future looks bright’), 5 points are given. The SISR-B contains four items that assess the key component process of recovery, i.e., finding hope, re-establishment of identity, finding meaning, and taking responsibility. Each item is rated on a 6-point Likert scale ranging from ‘disagree strongly (1 point)’ to ‘agree strongly (6 points)’. The summation of the scores of the four items was used as a primary outcome measure, and a higher score indicates a higher level of achieved recovery. The Japanese versions of the SISR-A and -B [19], which have been confirmed for their reliability and validity, were used in the present study.

## Clinical assessments

Clinical symptoms were assessed with the Positive And Negative Syndrome Scale (PANSS) [20]. The PANSS contains seven items for positive symptoms, seven for negative symptoms, and 16 for general psychopathology. Each item is rated from 1 to 7 points, and a higher score represents more severe psychopathology. In the current study, we used summation scores for positive symptoms, negative symptoms, and general psychopathology and the total sum of scores of all 30 items for the primary analyses; in addition, we used subscores for insight, depression, and anxiety from general psychopathology for the secondary analyses. The PANSS was administered by a trained rater (NM), who is a qualified nurse and not one of the ward staff. In cases where more information was needed for precise assessment, supervision and advice from the in-charge psychiatrists and ward staff were obtained to confirm the rating.

Self-efficacy is defined as ‘how well one can execute courses of action required to deal with a prospective situation’ [21]. The General Self-Efficacy Scale (GSES) [22] utilised in this study was developed to assess self-efficacy based on the model advocated by Bandura [23] and confirmed for its reliability and validity [22, 24]. The GSES is a self-reported measure that includes 16 items. In each item, a participant responds with ‘yes’ or ‘no’ to the questionnaire assessing self-efficacy, and 0 or 1 point is given to the response. The summation of scores was used for statistical analysis, and a higher score represents greater self-efficacy.

Self-esteem [25] was assessed with the Rosenberg Self-Esteem Scale (RSES). The RSES is a widely used self-reported measure that consists of 10 items, and each item is rated on a 4-point Likert scale ranging from ‘strongly disagree’ to ‘strongly agree’. One to 4 points were given according to each response, and the total score was used for statistical analysis. Higher scores represent higher self-esteem. The Japanese version of the RSES, which was confirmed for its reliability and validity [26], was administered in this study.

### Statistical analysis

Since the values of changes of indices for personal recovery were not normally distributed according to the Shapiro-Wilk test, we adopted non-parametric tests for statistical analyses in this study. As primary analyses, Wilcoxon signed-rank tests were administered for longitudinal comparisons between baseline and follow-up in terms of indices of personal recovery assessed with the QPR, RAS, SISR-A, and SISR-B; clinical symptoms assessed with PANSS positive symptoms, negative symptoms, general psychopathology, and total score; self-efficacy assessed with the GSES; and self-esteem assessed with the RSES. Spearman’s rank correlation tests were performed between longitudinal changes and baseline values in indices of personal recovery and between longitudinal changes in indices of personal recovery and baseline values or changes in PANSS positive symptoms, PANSS negative symptoms, PANSS general psychopathology, PANSS total score, GSES, and RSES. As secondary analyses, Spearman’s rank correlation tests were performed between longitudinal changes in indices of personal recovery and longitudinal changes in PANSS insight, PANSS depression, and PANSS anxiety; the duration of the current hospitalisation; the lifetime number of hospitalisations in psychiatric wards (including the current admission); and the estimated duration of illness from onset to the baseline assessment. The Mann-Whitney U test was performed for comparison of longitudinal changes in indices of personal recovery between participants who were voluntarily admitted at the beginning of the current hospitalisation and those who were involuntarily admitted. Statistical analyses were performed using IBM SPSS Statistics version 25.0 (IBM Corp., Armonk, NY, USA). Testing was two-tailed, and a significance level was set at 0.05. No correction was made for the probabilities of type I errors in order to avoid increasing the probabilities of type II errors due to low statistical power [27] in multiple pairwise correlation analyses.

## Results

### Basic data of the participants

Although all individuals who passed inclusion and exclusion criteria were considered eligible for participation in the study, around 30 individuals were judged to be inappropriate for participation by in-charge psychiatrists. In total, 47 individuals were permitted participation in the study by in-charge psychiatrists. Nine patients subsequently declined to participate in the study, and finally, 38 individuals provided informed consent to participate. Four participants (11%) dropped out of the study due to unexpected early discharge from the hospital. Three of those who dropped out were administered baseline assessments but did not undergo follow-up assessments, and one participant was not administered the baseline or follow-up assessments. Since these four participants were excluded from the analysis, 34 (89%) of the participants went through both baseline and follow-up assessments and were included in the analysis. Of the included participants, 22 (65%) were men, and 12 (35%) were women. The average age of the participants was 44.6 years (SD, 11.2; median, 45.0; range, 27–63 years). Thirty-three participants (97%) were diagnosed with schizophrenia, and one (3%) was diagnosed with schizoaffective disorder. Twenty-one (62%) participants were involuntarily admitted at the beginning of the current hospitalisation. The average duration of the current hospitalisation was 81.9 days (SD, 15.3; median, 85.0; range, 51–128 days). Baseline assessments were administered at 28.7 days after admission on average (SD, 12.3; median, 26.0; range, 8–57 days), and follow-up assessments were conducted at 77.3 days after admission on average (SD, 14.1; median, 78.0; range, 50–126 days). The interval between baseline and follow-up assessments was 48.6 days on average (SD, 15.4; median, 49.0; range, 21–98 days). The lifetime number of hospitalisations in psychiatric wards (including the current admission) was 4.0 on average (SD, 4.0; median, 2.0; range, 1–18). The estimated duration of illness from onset to the baseline assessment was 16.2 years on average (SD, 13.3; median, 12.0; range, 0.1–45.0 years).

### Longitudinal changes in personal recovery indices and other indices

The results of the Wilcoxon signed-rank tests for longitudinal comparisons between baseline and follow-up in terms of indices of personal recovery assessed with the QPR, RAS, SISR-A, and SISR-B; clinical symptoms assessed with PANSS positive symptoms, negative symptoms, general psychopathology, and total score; self-efficacy assessed with the GSES; and self-esteem assessed with the RSES are shown in Table 1.

**Table 1:** Longitudinal comparison of indices between baseline and follow-up

	Baseline		Follow-up		*P*-values
	Mean	SD	Mean	SD	
QPR	76.7	12.8	78.8	11.8	0.11
RAS	84.1	11.7	83.6	14.7	0.30
SISR-A	3.0	1.1	3.0	1.1	0.67
SISR-B	15.5	3.9	15.8	4.4	0.30
PANSS positive	17.5	3.7	13.2	4.7	< 0.001***
PANSS negative	18.4	3.5	14.6	4.4	< 0.001***
PANSS general	39.4	5.1	30.9	7.4	< 0.001***
PANSS total	75.4	11.0	58.7	15.1	< 0.001***
GSES	7.2	3.5	6.9	4.1	0.53
RSES	24.8	4.2	25.0	4.6	0.32

*GSES* General Self-Efficacy Scale, *PANSS* Positive And Negative Syndrome Scale, *QPR* Questionnaire about the Process of Recovery, *RAS* Recovery Assessment Scale, *RSES* Rosenberg Self-Esteem Scale, *SD* Standard deviation, *SISR-A* Self-Identified Stage of Recovery Part A, *SISR-B* Self-Identified Stage of Recovery Part B. Asterisks indicate significant differences (*P < 0.05, **P < 0.01, ***P < 0.001).

**Fig. 1:**
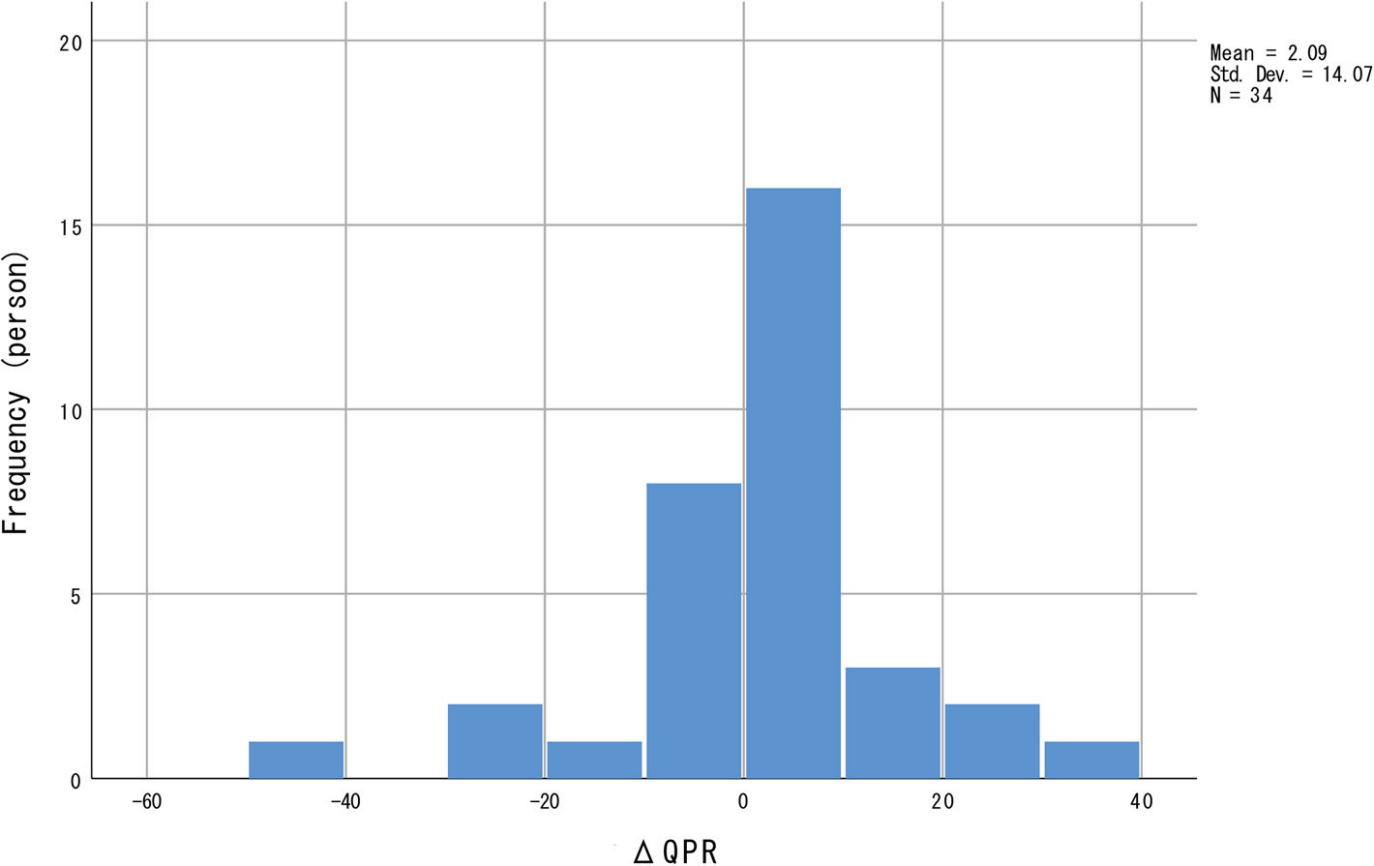
Distribution of changes in Questionnaire about the Process of Recovery (QPR) values. The frequency distribution is negatively skewed; most participants showed relatively small positive changes in QPR values, with a few showing greater negative changes

**Fig. 2:**
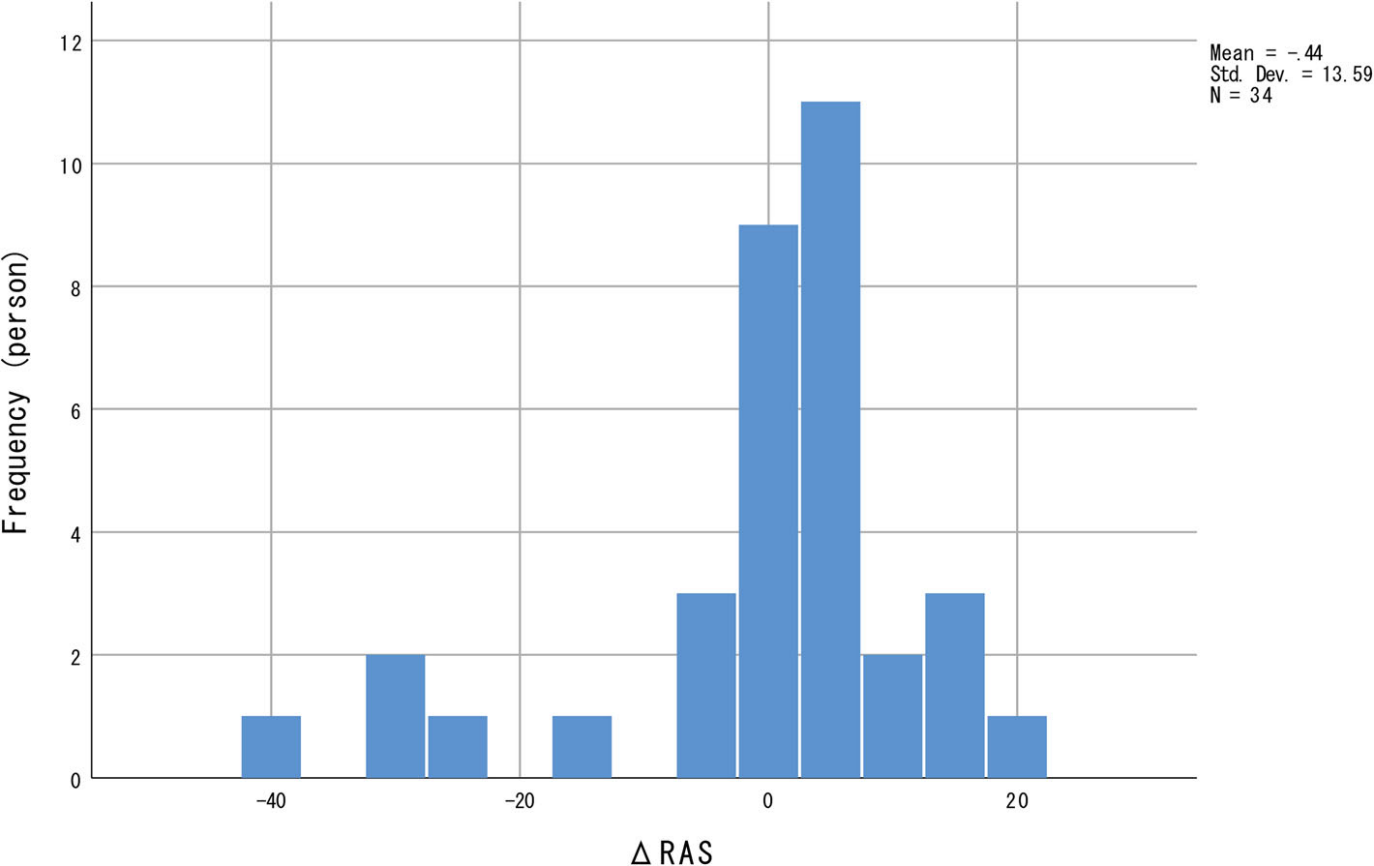
Distribution of changes in Recovery Assessment Scale (RAS) values. The frequency distribution is negatively skewed; most participants showed relatively small positive changes in RAS values, with a few showing greater negative changes

**Fig. 3:**
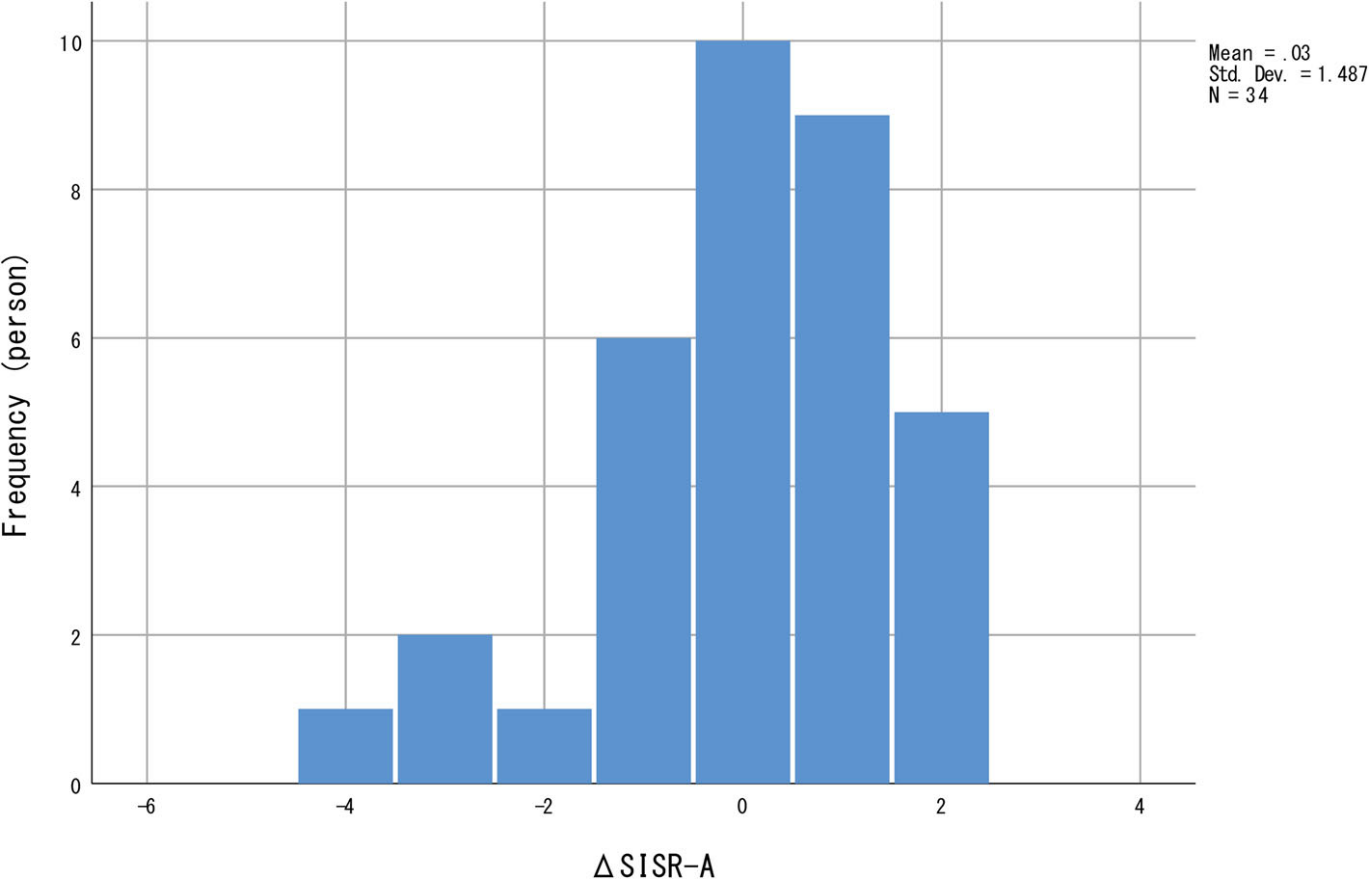
Distribution of changes in Self-Identified Stage of Recovery Part A (SISR-A) values. The frequency distribution is negatively skewed; most participants showed relatively small positive changes in SISR-A values, with a few showing greater negative changes

**Fig. 4:**
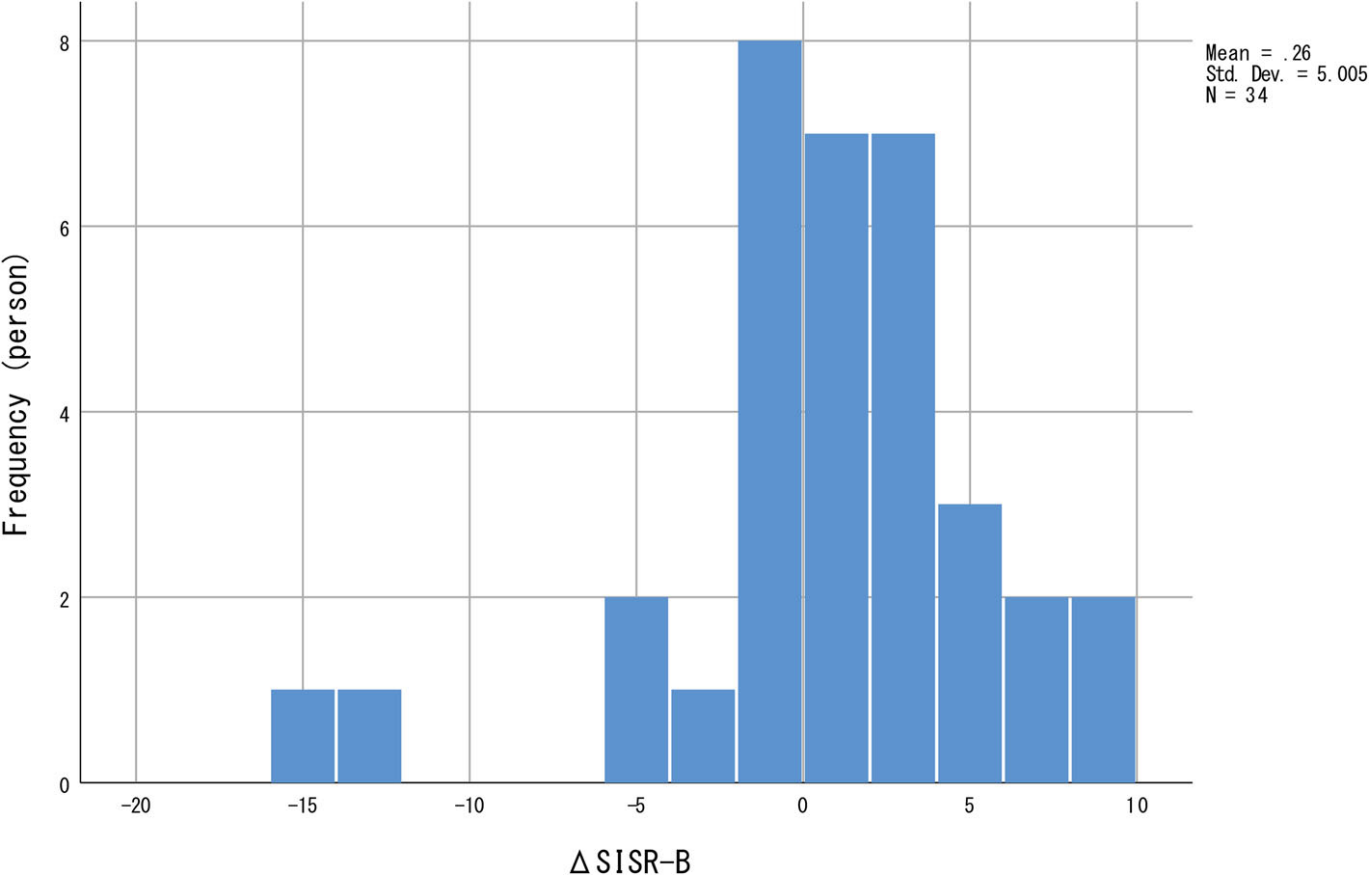
Distribution of changes in Self-Identified Stage of Recovery Part B (SISR-B) values. The frequency distribution is negatively skewed; most participants showed relatively small positive changes in SISR-B values, with a few showing greater negative changes

No significant longitudinal changes were observed in all four indices for personal recovery. The distribution of all four indices for personal recovery was negatively skewed (Figs. 1, 2, 3 and 4); most participants showed relatively small positive changes in indices for personal recovery, and a few participants had greater negative changes. Therefore, we subsequently qualitatively examined the characteristics of those who showed greater negative changes in indices for personal recovery and found that they were likely to show less amelioration in clinical symptoms during the hospitalisation.

There were significant improvements in positive symptoms, negative symptoms, general psychopathology, and total score of the PANSS during the hospitalisation. No significant change was found in the GSES and RSES during the hospitalisation.

### Correlations between changes in personal recovery indices and other indices

The results of Spearman’s rank correlation tests are shown in Table 2. A significant correlation was found between the positive change in the value of the RAS and improvement in negative symptoms. In addition, a correlation trend was found between the positive change in the value of the QPR and amelioration in negative symptoms. No significant correlation was found between the changes in indices for personal recovery and the changes in positive symptoms, general psychopathology, or total score assessed with the PANSS. Weak to moderate significant correlations were observed between the positive change in the GSES and the positive changes in the indices for personal recovery assessed with the QPR, RAS, and SISR-A, and a correlation trend was found between the positive change in the GSES and that in the SISR-B. A moderate significant correlation was observed between the positive change in the RSES and the SISR-B, and a correlation trend was found between the positive change in the RSES and the positive changes in the QPR and RAS.

**Table 2:** Correlations between changes in personal recovery indices and other indices

	QPR, change	RAS, change	SISR-A, change	SISR-B, change
PANSS positive, change	−0.09	− 0.15	0.22	− 0.08
PANSS negative, change	−0.31	− 0.35*	0.13	− 0.18
PANSS general, change	−0.12	− 0.24	0.07	− 0.22
PANSS insight, change	−0.26	− 0.02	− 0.19	− 0.01
PANSS depression, change	− 0.01	− 0.25	−0.04	− 0.20
PANSS anxiety, change	0.08	−0.03	0.06	−0.01
PANSS total, change	−0.19	−0.25	0.15	−0.21
GSES, change	0.49**	0.39*	0.37*	0.32
RSES, change	0.29	0.29	0.19	0.47**
QPR, baseline	−0.42*	−0.30	−0.13	− 0.23
RAS, baseline	−0.12	− 0.37*	0.16	− 0.11
SISR-A, baseline	−0.31	− 0.31	−0.67**	− 0.24
SISR-B, baseline	−0.40*	− 0.36*	−0.12	− 0.47**
PANSS positive, baseline	−0.25	− 0.12	−0.03	− 0.14
PANSS negative, baseline	0.02	0.05	0.00	−0.13
PANSS general, baseline	−0.19	−0.04	− 0.03	−0.06
PANSS total, baseline	−0.13	−0.01	0.04	−0.06
GSES, baseline	−0.13	−0.16	− 0.10	0.08
RSES, baseline	−0.26	−0.33	0.05	−0.28
Duration of hospitalisation	0.18	−0.14	−0.12	0.15
Numbers of hospitalisations	−0.16	−0.14	− 0.06	−0.10
Duration of illness	−0.01	−0.10	0.15	0.06

*GSES* General Self-Efficacy Scale, *PANSS* Positive And Negative Syndrome Scale, *QPR* Questionnaire about the Process of Recovery, *RAS* Recovery Assessment Scale, *RSES* Rosenberg Self-Esteem Scale, *SISR-A* Self-Identified Stage of Recovery Part A, *SISR-B* Self-Identified Stage of Recovery Part B. The values show Spearman’s rank correlation coefficients (rho), and asterisks indicate significant correlations (*P < 0.05, **P < 0.01, ***P < 0.001)

In all four indices for personal recovery, weak to strong significant negative correlations were found between baseline values and longitudinal changes during the hospitalisation. There were no significant correlations between changes in the indices for personal recovery and baseline values for any clinical symptoms or the GSES. A negative correlation trend was observed between the positive change in the RAS and the baseline value of the RSES.

As secondary analyses, correlations between changes in indices for personal recovery and other indices were examined (Table 2). No significant correlations were found between changes in indices for personal recovery and the changes in insight, depression, or anxiety as assessed with the PANSS; the duration of the current hospitalisation; the lifetime number of hospitalisations in psychiatric wards (including the current admission); or the estimated duration of illness from onset to the baseline assessment. The Mann-Whitney U test showed no significant difference in longitudinal changes in indices of personal recovery between participants who were voluntarily admitted at the beginning of the current hospitalisation and those who were involuntarily admitted.

## Discussion

Many studies have demonstrated a cross-sectional relationship between clinical recovery and personal recovery in patients with psychotic disorders, and a meta-analysis indicated that severity in both positive and negative symptoms is negatively correlated with personal recovery [5]. Accordingly, we hypothesised that indices for personal recovery in individuals with psychotic disorders would be positively changed through hospitalisation because their clinical symptoms would improve through the care received during hospitalisation. To the best of our knowledge, this study is the first to investigate whether indices for personal recovery would change through hospitalisation in individuals with psychotic disorders. Unexpectedly, the results revealed that the indices for personal recovery did not change through hospitalisation, although clinical symptoms significantly improved. In addition, longitudinal changes in indices for personal recovery were partly correlated with amelioration in negative symptoms and enhancement in self-efficacy and self-esteem. Since distributions of all four indices for personal recovery were negatively skewed, the lack of longitudinal changes in indices for personal recovery in this cohort did not indicate that indices for personal recovery in each individual did not change at all; it is possible that these indices could change longitudinally to some degree, and that there could be individuals with both positive and negative changes in indices for personal recovery in this cohort. Correlation analyses showed that individuals with positive changes in indices for personal recovery were likely to have ameliorated negative symptoms, self-efficacy, and self-esteem.

A previous meta-analysis demonstrated that the magnitude of cross-sectional correlation was greater between degrees of personal recovery and severity of negative symptoms (correlation coefficient, − 0.26) than with the severity of positive symptoms (correlation coefficient, − 0.14) [5]. The present results showed that such a relationship could be observed longitudinally, which is consistent with the findings in the meta-analysis with cross-sectional studies. The RAS, which was found to have a longitudinal relationship with negative symptoms, contains such factors as the willingness to ask for help and reliance on others [15]. Severe negative symptoms could cause withdrawal in individuals with psychotic disorders and hamper connectedness with others. Therefore, amelioration in negative symptoms through a hospitalisation might have enhanced those attitudes, such as willingness to ask for help or reliance on others, and therefore has a longitudinal relationship with the changes in degrees of personal recovery assessed by the RAS.

Additionally, in line with the previous findings [6, 7], the current study revealed longitudinal relationships between degrees of personal recovery and self-efficacy or self-esteem. Self-efficacy is an expectancy of how well one can execute courses of action required to deal with the prospective situation [21]. Thus, individuals with high self-efficacy would be likely to have a self-certainty to achieve something. Self-efficacy could be related to key factors of personal recovery such as ‘hope and optimism about the future’ [2]. Therefore, individuals who gained high self-efficacy during a period of hospitalisation were likely to show a positive change in indices for personal recovery. Similarly, self-esteem, which is also an aspect of the positive appraisal of the self and could have common features with the concept of personal recovery, might have caused the longitudinal relationship between personal recovery and self-esteem observed in the present study. These findings may imply that enhancing the positive appraisal on self, such as self-efficacy and self-esteem, should be important to achieve personal recovery for individuals with psychotic disorders through hospitalisation.

In all four indices employed in this study, each degree of positive change in personal recovery was negatively correlated with each baseline value for personal recovery. Although pre-admission values of indices for personal recovery were not attained in this study, the possibility of personal recovery in individuals with psychotic disorders might have been hampered before admission in a psychiatric ward due to the deterioration of symptoms affecting the life and negative perspective for the future. Therefore, such individuals could get benefits from the care offered during hospitalisation and potentially have more positive changes in personal recovery.

Since the participants in the current study were all inpatients, it is possible that their characteristics of personal recovery and the related factors could be different from those of outpatients. Nevertheless, in the study by Chiba et al. [14] that included both inpatients and outpatients and used the Japanese version of the RAS, the RAS values were 81.0 (SD, 16.1) in the inpatients and 83.5 (SD, 15.3) in the outpatients. Although there are some differences in the background of the participants between this and the current study, for example, the study of Chiba et al. [14] included many individuals diagnosed with disorders other than schizophrenia, the findings are consistent between the two studies.

The length of hospitalisation of the participants in the current study was relatively moderate. Although the duration of hospitalisation was not correlated with the magnitude of changes in indices for personal recovery in the current study, it is possible that the results might have been different if the participants had been recruited from the ward providing more acute treatment or long-term care. Chronicity in the individuals would also affect the change of personal recovery. Most of the current participants had a relatively chronic illness, and only a small number of participants were experiencing their first episode of psychosis. Although the lifetime number of hospitalisations in psychiatric wards and the estimated duration of illness were not correlated with the magnitude of changes in indices for personal recovery among the current participants, whether individuals with early psychosis are likely to show greater changes in indices for personal recovery should be confirmed in the future studies. In addition, whether inpatients with more severe or less severe symptoms show similar findings should also be examined. The existing research examining personal recovery among inpatients has been sparse; one study that recruited inpatients with schizophrenia showed more severe positive symptoms as assessed with the PANSS (average, 26.6; SD, 14.6) and a greater magnitude of the index for personal recovery as assessed with the RAS (average, 89.2; SD, 17.6) than did the current study [28].

Lack of positive changes in the indices for personal recovery through hospitalisation in the current study raises the possibility that care in a hospital could have precluded the achievement of personal recovery for the individuals. More than half of the participants in the current study were involuntarily admitted to the ward. For the achievement of personal recovery, it has been suggested that the process of decision-making should be kept as close to the service users as possible [3], and coercion should be avoided as much as possible [8]. The possibility of personal recovery in the current participants might have been deprived due to the limitations of autonomy and self-decision-making caused by coercion during hospitalisation. Moreover, the staff in the units were not trained in recovery-oriented care, and the participants were not offered care based on a recovery-centred approach, which might have partly precluded the sufficient promotion of personal recovery in the current participants during their hospitalisation period.

There are several limitations to this study. The sample size in this study was relatively small. For 34 participants, if the significance level is set at 0.05 and the statistical power is set at 0.8, the needed effect size is then calculated to be 0.51 (G*power 3.1.9.7). The obtained effect sizes for the results of Wilcoxon signed-rank tests comparing longitudinal changes between baseline and follow-up in terms of indices of personal recovery assessed with the QPR, RAS, SISR-A, and SISR-B were 0.33, 0.17, 0.07, and 0.17, respectively. Although the lack of significant longitudinal changes in indices for personal recovery in the current sample could be due to the small observed effect sizes, it could also be affected by underpowered tests caused by the small sample size. Thus, the current findings should be considered preliminary, and further studies with a larger sample size are needed to confirm these findings.

There are additional limitations to this study. Individuals with severe symptoms or who had difficulty in communication were not likely to be recruited in the current study. The duration of illness was different among the participants. The reason for admission for each participant was also different and was not always due to the deterioration of symptoms. The interval period between baseline and follow-up was also different amongst participants. Moreover, because this study was a pre-post study involving only a single group without a control group, the offered interventions could be different among the participants.

There is also a limitation related to the assessment of personal recovery. The definition of personal recovery has not been fully established, and personal recovery has been interpreted in different ways. Similarly, various scales have been used to measure personal recovery. As shown in the current study, interpretation of the difference in various indices for personal recovery is often difficult. Further, personal recovery may include similar concepts that have been defined as other terms, i.e. hope, quality of life, empowerment, or well-being [2, 4, 5, 29]. Personal recovery should be strongly associated with the individual context of one’s own life. Therefore, validity could be determined by whether scales for personal recovery could appropriately assess one’s personal recovery associated with individual aspects [30].

In this study, we investigated several factors that could be related to the changes in personal recovery. Nevertheless, there are other candidate factors related to the changes in personal recovery based on previous findings with cross-sectional relationships [31]. Affective symptoms have been proved to have stronger effects on personal recovery than positive or negative symptoms [5]. In addition, neurocognition [32] and insight [28, 33] have been shown to be related to personal recovery. Although insight, depressive, or anxiety symptoms as assessed with the PANSS were not correlated with changes of indices for personal recovery in the current study, these assessments were performed using single items of the PANSS. These preliminary findings should be confirmed using more specific measures in future studies. Recovery of social functioning, sometimes called ‘functional recovery‘, which is separated from ‘clinical recovery’ [34], has been demonstrated to be correlated with personal recovery [30, 35]. The factors above were not examined in this study but might also have longitudinal relationships with changes in personal recovery occurring during hospitalisation.

## Conclusions

Despite the aforementioned limitations, we have found important longitudinal relationships between changes in personal recovery and amelioration of negative symptoms and enhancement of self-efficacy and self-esteem in individuals with psychotic disorders during hospitalisation. Although findings of personal recovery in inpatients are relatively sparse, implementation of a recovery-oriented approach in an acute psychiatric ward has been demonstrated to enhance recovery orientation of the staff [36] and attitudes towards recovery and self-assessment of the recovery process of the inpatients [8]. Another study has shown that recovery-promoting competencies of mental health providers are positively associated with changes in personal recovery of involuntarily admitted individuals [37]. The current findings suggest that such recovery-promoting approaches could benefit from efforts enhancing both the self-efficacy and self-esteem of the individuals. Therefore, the ward staff should minimise coercion and maximise autonomy and self-decision-making with inpatients with psychotic disorders. In addition, the effectiveness of specific interventions on the enhancement of personal recovery, including illness management and recovery [38–42], recovery programs including benefit finding [43], cognitive-behavioural therapy [44–47], Wellness Recovery Action Planning [48], and use of digital devices [49, 50], has been reported in recent years. In the future, the effectiveness of such specific interventions in inpatients should be examined.

## Data Availability

Most data generated or analysed during this study are included in this published article. Additional data not shown in this article will be available from the corresponding author on reasonable request.

## Abbreviations

GSES: General Self-Efficacy Scale
PANSS: Positive And Negative Syndrome Scale
QPR: Questionnaire about the Process of Recovery
RAS: Recovery Assessment Scale
RSES: Rosenberg Self-Esteem Scale
SISR-A, SISR-B: Self-Identified Stage of Recovery Part A, Part B
